# Aspergillose sterno-costale après angioplastie coronaire: à propos d’un cas

**DOI:** 10.11604/pamj.2022.42.226.32841

**Published:** 2022-07-21

**Authors:** Ahd Ouladlahsen, Sanaa Jebbar, Laila Benkiran, Latifa Marih, Mustapha Sodqi, Soussi-Abdellaoui Maha, Zouhair Chefchaouini, Brahim Idali, Chaoui Zouhair

**Affiliations:** 1 Faculté de Médecine et de Pharmacie, Université Hassan II, Casablanca, Maroc,; 2 Service des Maladies Infectieuses, Centre Hospitalier Universitaire Ibn Rochd, Casablanca, Maroc,; 3 Laboratoire de Pathologie Bourgogne, Casablanca, Maroc,; 4 Service de Parasitologie Mycologie, Centre Hospitalier Universitaire Ibn Rochd, Casablanca, Maroc,; 5 Centre de Chirurgie Cardio-Vasculaire Dar Salam, Casablanca, Maroc,; 6 Radiologie Quartier des Hôpitaux, Casablanca, Maroc

**Keywords:** *Aspergillus*, diabète, ostéomyélite, sternum, cas clinique, Aspergillus, diabetes, osteomyelitis, sternum, case report

## Abstract

L´aspergillose sternale est extrêmement rare. Parmi tous les cas d´aspergillose invasive rapportés dans la littérature, l´incidence de l´ostéomyélite est inférieure à trois pour cent. L´aspergillose survient préférentiellement sur un terrain d´immunodépression. La présentation clinique et radiologique est peu spécifique. La contamination se fait essentiellement par inhalation de spores, mais elle peut aussi atteindre directement une zone vulnérable après un acte médical. Le diagnostic est souvent difficile à faire et peut nécessiter plusieurs semaines. Surtout si le médecin ne pense pas à un Aspergillus. Le diagnostic positif est orienté par l´imagerie mais sa confirmation est anatomopathologique et/ou mycologique. Le pronostic dépend essentiellement de la précocité d´administration du traitement. Nous présentons un patient de 63 ans, diabétique, ayant une ostéomyélite à Aspergillus de localisation sterno-costale survenue après une angioplastie coronaire.

## Abstract (en)

Sternal osteomyelitis due to aspergillosis is extremely rare. Among all cases of invasive aspergillosis reported in the literature, the incidence of osteomyelitis is less than 3%. Aspergillosis mainly affects immunosuppressed patients. Clinical and radiological manifestation is nonspecific. Contamination is primarily caused by inhaling spores, but it can also directly reach a vulnerable area after medical procedure. Diagnosis is often difficult and may take several weeks, in particular when aspergillosis is not suspected. Positive diagnosis is based on imaging tests but it is confirmed by anatomopathological and/or mycological examinations. Prognosis mainly depends on early administration of treatment. We here report the case of a 63-year-old diabetic patient with sternocostal osteomyelitis due to Aspergillus occurred after coronary angioplasty.

## Introduction

En cardiologie interventionnelle, l´angioplastie coronaire est devenue une technique de routine. Néanmoins, elle constitue un geste invasif pourvu de complications graves essentiellement vasculaires (saignement, hématome, pseudo-anévrisme, fistule artério-veineuse, ischémie de membre) mais aussi infectieuses dues principalement à des bactéries mais aussi à des agents fongiques dont l´*Aspergillus*. Parmi les localisations rapportées, il y a l´ostéomyélite à *Aspergillus* de la cage thoracique qui est une entité relativement rare, avec un taux d´incidence de seulement 9% [1]. C´est une complication exceptionnelle d´un acte non chirurgical comme l´angioplastie. L´aspergillose sternale est extrêmement rare. Parmi tous les cas d´aspergillose invasive rapportés dans la littérature, l´incidence de l´atteinte sternale est inférieure à 3% [2]. Nous rapportons ici le premier cas, à notre connaissance, d´un patient qui a développé une ostéomyélite sterno-costale à *Aspergillus* dans les suites d´une double angioplastie sans qu´il y est une atteinte pulmonaire ou cardiaque associée.

## Patient et observation

**Information du patient**: M. BD Gabonais, âgé de 63 ans, suivi pour un diabète type 2 sous insuline, un asthme stable et une hypertension artérielle (HTA). Il a été traité initialement, en dehors du Maroc, le 20 septembre 2020 par double angioplastie de la coronaire droite et de la circonflexe avec un bon résultat tout en gardant des douleurs précordiales puis il a été adressé au Gabon. En janvier 2021, il a été transféré au Maroc devant la persistance des précordialgies et l´apparition des douleurs thoraciques irradiant vers le dos d´intensité croissante.

**Résultats cliniques**: l´examen clinique à l´admission avait trouvé un patient conscient, bien orienté dans le temps et dans l´espace, apyrétique, une glycémie capillaire à 1,40 g/l, une tension artérielle à 120/70 mmHg, une fréquence cardiaque à 86 batt/min et la saturation à l´air libre à 97%, pas d´œdème des membres inférieurs, mollets libres et signe de Homans négatif, pas de souffle cardiaque et les conjonctives étaient normo-colorées. Il avait des râles crépitants à l´auscultation pulmonaire et des douleurs exquises déclenchées par la palpation du sternum et des espaces intercostaux.

**Démarche diagnostique**: le bilan biologique du 09/01/2021 avait montré les résultats suivants: troponine Ic <0,10 ng/ml, D-dimères à 1250 ng/ml, hémoglobine à 8,8 g/dl, leucocytes à 15.840/ mm^3^, les plaquettes à 541 000/mm^3^, protéine C-réactive (CRP) à 77,63 mg/l, procalcitonine à 0,05 ng/ml, bilan hépatique normal, un taux de prothrombine (TP) à 100%, un bilan lipidique normal, urée à 0,32 g/l et créatinine à 11,36 mg/l, glycémie à 1,68 g/l, taux d’antigène prostatique spécifique (PSA) à 3,250 ng/mn, la férritinémie à 438,20 ng/ml. L´examen direct des expectorations avait isolé un cocci gram positif avec culture négative. L´examen cytobactériologique des urines était stérile.

A l´électrocardiogramme, le rythme était régulier sinusal à 90 batt/mn avec un microvoltage et un segment ST raide en DI-Avl. L´échographie cardiaque avait objectivé une cardiopathie hypertensive à minima, bonne fonction systolique, fraction d'éjection ventriculaire gauche (FEVG) à 66%, les pressions de remplissage étaient basses, absence de valvulopathie mitro-aortique significative, les pressions pulmonaires étaient normales. Une coronarographie avait montré une sténose significative de l´artère interventriculaire antérieure proximale englobant l´origine de la première diagonale (lésion de bifurcation), absence de resténose de l´artère circonflexe proximale au niveau du site d´implantation du stent actif, sténose significative de l´artère circonflexe moyenne englobant l´origine de la seconde marginale (lésion de bifurcation), absence de resténose de l´artère interventriculaire postérieure au niveau du site d´implantation du stent actif, sténose significative de l´artère rétro-ventriculaire postérieure. Au scanner thoracique, nous avons noté un épanchement pleural bilatéral de faible abondance, plus marqué du côté droit, un foyer de condensation alvéolaire au niveau du segment postéro-basal du lobe inférieur droit, évoquant un collapsus passif, atélectasie en bande lobaire inférieure droite, épaississements septaux lobaires inférieurs gauches avec absence de bronchectasie ou de bulle d´emphysème, aspect normal du médiastin sans adénomégalie notable, opacification normale des vaisseaux médiastinaux, notamment absence de signe de dissection aortique. L´aspect était en faveur d´une pleuro-pneumopathie basale. Un traitement à base d´amoxicilline-acide clavulanique a été démarré mais l´évolution était défavorable ce qui a motivé un changement par les fluoroquinolones et les céphalosporines de 3^e^ génération mais sans aucune amélioration avec persistance des douleurs thoraciques et l´augmentation des taux de leucocytes et de la CRP. La recherche de Bacille de Koch (BK) dans les expectorations était négative.

**Figure 1:**
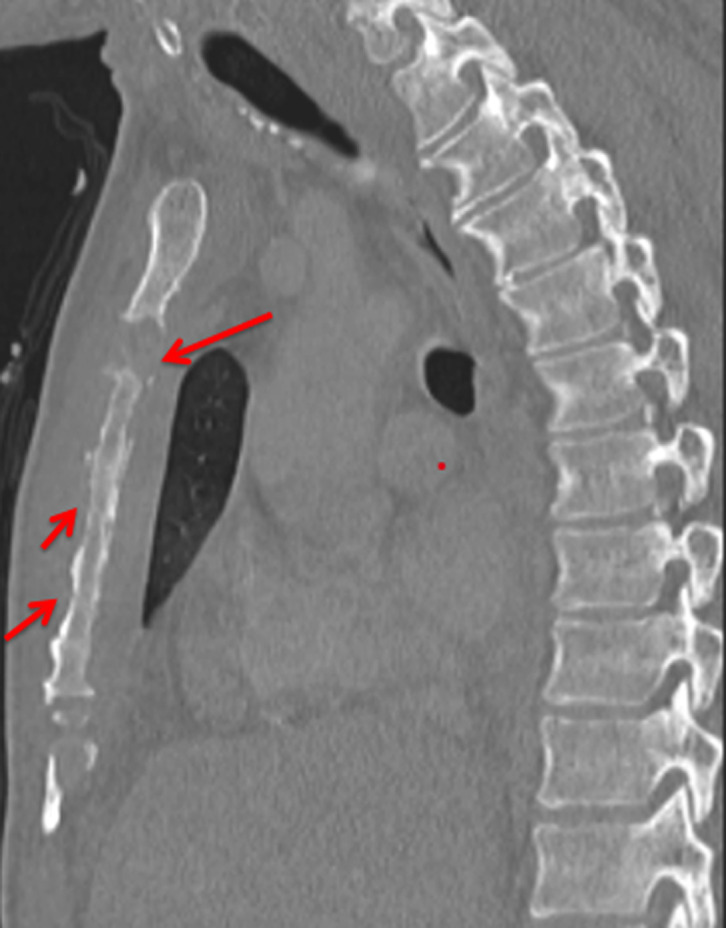
atteinte osseuse lytique intéressant le sternum et prédominant au niveau de la 2^e^ pièce sternale avec multiples lyses des arcs antérieur avec réaction périostée

**Figure 2:**
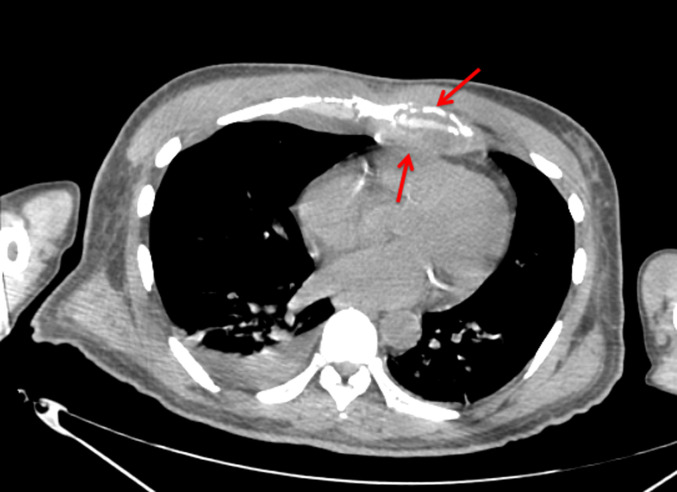
lyse osseuse de l’extrémité interne de l’arc costal antérieur avec collections pariétales hypodenses

**Figure 3:**
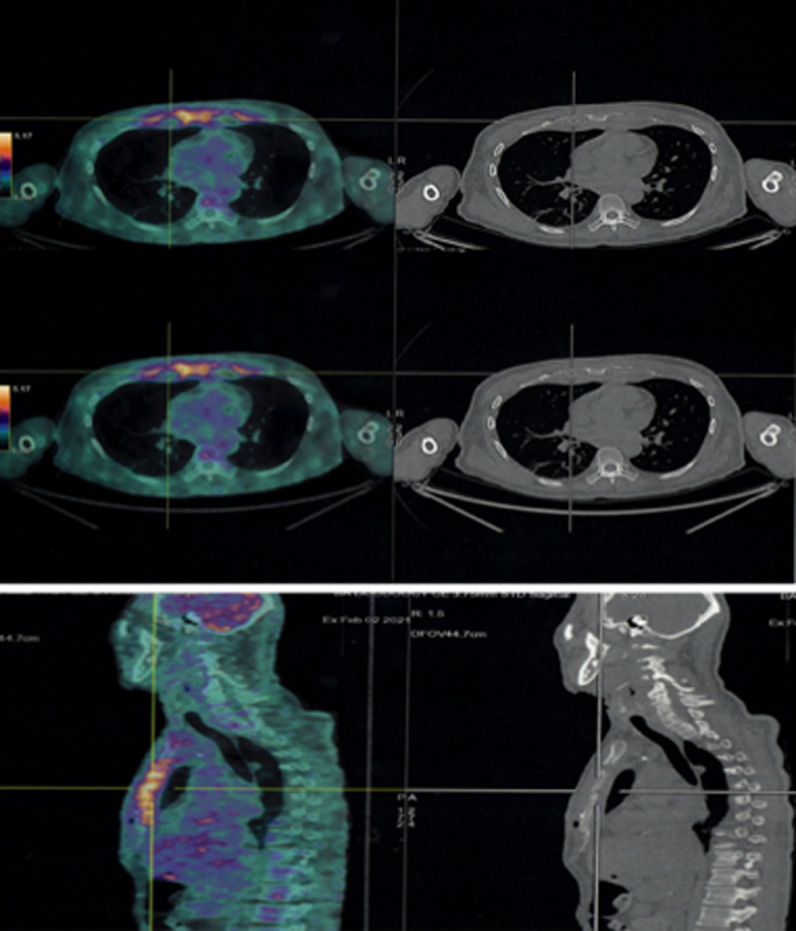
lésions ostéolytiques hyper métaboliques bilatérales de la quasi-totalité des jonctions chondrosternales aves aspect grignoté du bord latéral du manubrium et du corps sternal

La scintigraphie osseuse avait mis en évidence des lésions sternales, costales des jonctions stérnocostales bilatérales majorées à gauche. L´aspect ostéolytique a été rattaché à un processus prolifératif dont la nature secondaire n´a pas été exclue. Le scanner thoraco-abdomino-pelvien réalisé le 22/01/2021 avait montré une atteinte osseuse lytique intéressant le sternum et prédominant au niveau de la 2^e^ pièce sternale qui est le siège d´une lyse étendue sur une hauteur de 2 cm environ (Figure 1). Il s´est associé une atteinte érosive des arcs costaux antérieurs aves collections pariétales hypodenses (Figure 2) notamment en regard de l´arc antérieur de la 3^e^ côte droite 35x19mm et de l´arc antérieur de la 4^e^ côte gauche 45x24mm, une condensation alvéolaire basipulmonaire droite, d´allure infectieuse, une formation polypoïde de la région fundique de la vésicule biliaire de type tissulaire nettement rehaussée après injection de produit de contraste évaluée pratiquement à 22 mm de grand axe. Pancréas atrophique avec calcifications et ectasie distale du canal de Wirsung pouvant être dans le cadre d´une pancréatite chronique. Une échographie thyroïdienne réalisée le 01/02/2021 avait montré des kystes thyroïdiens sans caractère suspect, classé (*European Thyroïd Imaging-Reporting and Data System*) 2. Une tomographie par émission de positons (PET-scan) réalisé le 02/02/2021 avait montré des lésions ostéolytiques hypermétaboliques bilatérales de la quasi-totalité des jonctions chondrosternales aves aspect grignoté du bord latéral du manubrium et du corps sternal (Figure 3).

**Figure 4:**
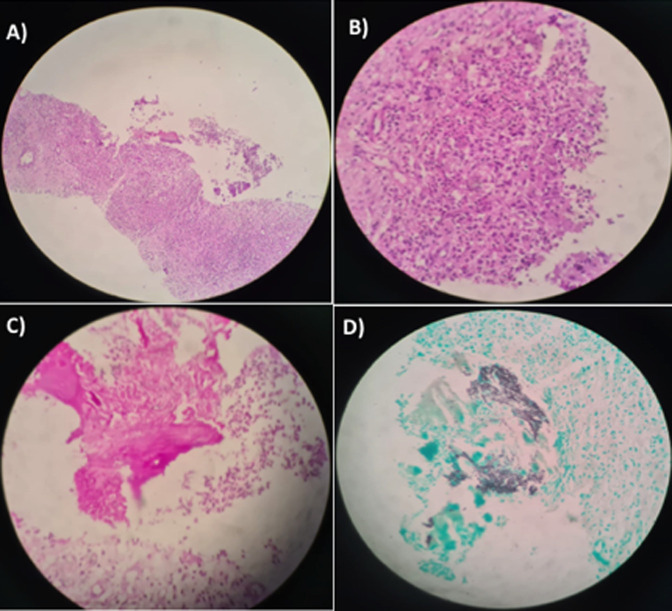
étude anatomopathologique, A) tissu de granulations inflammatoires polymorphes avec lyse osseuse; B) ébauche de granulome épithélioïde et gigantocellulaire sans nécrose; C) filaments mycéliens enchevêtrés sur l’hématéine éosine; D) coloration du Gomori Grocott mettant en évidence des filaments mycéliens septés et branchés à angle aigu compatible avec des filaments aspergillaires

Au vu de ces données, le staff medico-chirurgical avait conclu à la nécessité de réaliser une cholécystectomie emportant le polype intravésical et une biopsie sternale. La biopsie parasternale réalisée le 05/02/2021 avait objectivé des lésions d´ostéite chronique, granulomateuse sans nécrose caséeuse associées à la présence de filaments mycéliens septés, pouvant correspondre à des filaments aspergillaires (Figure 4A,B,C,D). La culture du liquide intralésionnel avait mis en évidence des colonnies aspergillaires. La sérologie aspergillaire était négative. Le diagnostic de l´aspergillose invasive a été prouvé selon les critères de l’Organisation Européenne pour la Recherche et le Traitement du Cancer (EORTC) avec une preuve histopathologique formelle et une culture mycologique positive. La sérologie VIH était négative, le dosage des sous populations lymphocytaires n´avait pas montré d´anomalies et le dosage pondéral des immunoglobulines était normal avec (IgA =2,47 g/l; IgG=11,53 g/l; IgM=1,33 g/l). Par ailleurs, le test dihydrorhodamine (DHR) avait montré une réponse faible des polynucléaires neutrophiles (PNN) aux différentes stimulations plus marqué avec le phorbol-myristate-acetate (PMA), les index de stimulation des PNN activés à *E. Coli* et PMA étaient respectivement de 37% et 16% avec des PNN à 50% et PNN activés à 85%.

**Intervention thérapeutique**: un traitement à base de voriconazole a été prescrit le 26 février 2021, et maintenu pendant 8 mois, à raison de 6mg/kg/à j1 puis 3mg/kg deux fois par jour à partir de j2 avec une surveillance de signes cliniques et biologiques d´intolérance. Un traitement antalgique à base de tramadol 100mg LP et de morphine à la demande a été prescrit.

**Figure 5:**
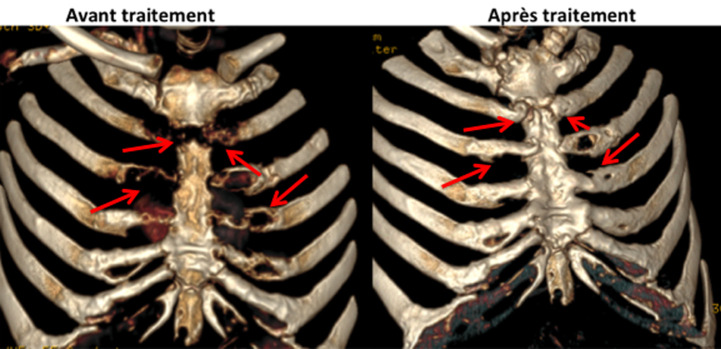
régression complète des collections liquidiennes abcédées en regard des arcs costaux antérieurs et des articulations costo-sternales de 2^e^ à la 4^e^ côte droite et de la 2^e^ à la 5^e^ côte gauche

**Figure 6:**
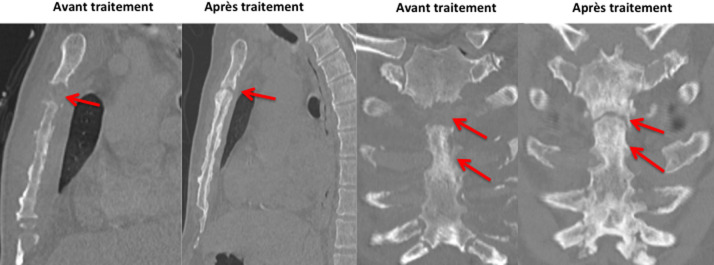
régression des érosions osseuses du sternum et de la lyse de la 2^e^ pièce sternale avec des phénomènes de reconstruction osseuse et d´épaississement périosté

**Suivi**: l´évolution était favorable au plan clinique et radiologique avec au scanner thoracique de contrôle du 28/09/2021, il y avait une régression complète des collections liquidiennes abcédées en regard des arcs costaux antérieurs et des articulations costo-sternales de la 2^e^ à la 4^e^ côte droite et de la 2^e^ à la 5^e^ côte gauche (Figure 5) associée à une régression également des érosions osseuses du sternum et de la lyse de la 2^e^ pièce sternale avec des phénomènes de reconstruction osseuse et d´épaississement périosté (Figure 6).

**Perspective du patient**: pendant son hospitalisation, il était satisfait des soins prodigués et de l´amélioration de son état clinique.

**Consentement du patient**: le consentement éclairé a été obtenu pour utiliser les données iconographiques.

## Discussion

L´*Aspergillus* est un champignon filamenteux ubiquitaire constamment retrouvé dans l´environnement y compris dans les établissements hospitaliers. La contamination est essentiellement respiratoire par inhalation de spores suspendues dans l´atmosphère [3]. L´aspergillose est une affection rare survenant particulièrement chez les patients immunodéprimés. Dans cette population, l´*Aspergillus* est le deuxième agent responsable de mycoses profondes, après *Candida albicans* [4]. La corticothérapie, à posologie élevée et prolongée, la neutropénie, la granulomatose septique chronique, l´infection à VIH et la greffe d´organe solide ou de cellules souches, le traitement par antagonistes du facteur de nécrose tumorale (TNF)-alpha et le diabète sont reconnus comme facteurs favorisants les aspergilloses [5]. Le facteur de risque retrouvé chez ce patient était le diabète mal équilibré, cependant, l´altération fonctionnelle des PNN fait suspecter chez lui une granulomatose septique chronique. L´*Aspergillus* touche avec prédilection les poumons où l´atteinte peut aller d´une mycose localisée à des infections invasives d´évolution souvent mortelle. Cependant, les ostéomyélites aspergillaires sont possibles mais rares et occupent la 4^e^ place après l´atteinte des poumons, des sinus et du cerveau. Elles représentent moins de 3% des cas d´aspergillose invasive [4]. L´ostéomyélite peut survenir par inoculation directe (traumatique ou iatrogène), par dissémination hématogène ou par contiguïté [6]. Les sites osseux préférentiels de l´aspergillose sont les vertèbres, les côtes, le sternum et le crâne [6]. L´ostéomyélite sternale est rare, la plupart des cas sont post-traumatiques ou post-chirurgicaux. Plusieurs cas d´ostéomyélite sternale à *Aspergillus* ont été rapportés dans la littérature, survenant essentiellement après sternotomie [7].

Le diagnostic de l´aspergillose a été retenu après 4 mois d´évolution. L´hypothèse proposée était un ensemencement hématogène du tissu osseux à partir des stents infectés [8]. La présentation clinique était atypique, la douleur osseuse aggravée par l´inspiration profonde et la mastication constituait le maître symptôme. La fièvre était absente et l´état général était altéré ce qui est concordant avec la littérature [6]. Le diagnostic est généralement tardif vu l´évolution prolongée de la maladie. La durée moyenne de diagnostic d´ostéomyélite fongique est de 32 semaines [9]. Rien n´explique cette latence variable mais l´immunité de l´hôte et la taille de l´inoculum pourraient jouer un rôle. Chez notre patient, le diagnostic était évoqué devant la persistance de la douleur, l´augmentation des taux des marqueurs biologiques de l´inflammation, la survenue d´une tuméfaction des parties molles en regard du stérnum et la non amélioration sous traitement antibiotique à large spectre.

L´imagerie par résonance magnétique (IRM) osseuse constitue l´examen de référence dans les ostéomyélites aspergillaires. La réalisation de la tomographie par émissions de positrons au 18F-fluorodésoxyglucose/tomographie (FDG-PET/CT scan) peut être d´une très grande utilité pour le diagnostic, cependant, il reste un traceur non spécifique qui peut être rencontré dans d´autres infections. Son rôle majeur est de révéler l´étendue de l´atteinte, ainsi que le suivi sous thérapies antifongiques. Cet examen avait montré des lésions hypermétaboliques ostéolytiques bilatérales des jonctions chondrosternales sans autres sites d´hypermétabolisme suspect. La biologie est non spécifique au cours des infections aspergillaires, le syndrome inflammatoire est le plus souvent noté et les hémocultures sont généralement stériles. Le dosage du galactomannane, qui est un outil diagnostique sensible, n´est pas de pratique courante, non réalisé pour ce patient. Peu de données sont disponibles concernant les dosages du galactomannane dans le diagnostic de l´ostéomyélite fongique et des infections articulaires. Selon les études, seuls dans 4 cas sur 47 où la galactomannanantigénémie était positive [10-13]. Les méthodes de détection d´acide désoxyribonucléique (ADN) d´*Aspergillus* par *polymerase chain reaction* (PCR) ne sont pas encore standardisées. La sérologie aspergillaire est d´interprétation difficile et de faible spécificité, elle est de moins en moins utilisée en pratique clinique. La sérologie aspergillaire de notre patient était négative. La confirmation peut être microbiologique par l´examen direct et la culture des prélèvements osseux et/ou du pus ou anatomopathologique par la mise en évidence de filaments mycéliens septés “de type aspergillaire”. De ce fait, la certitude diagnostique d´une ostéomyélite aspergillaire nécessite une histologie positive associée à une culture positive dans un contexte clinique compatible. L´espèce la plus fréquemment retrouvée est *Aspergillus fumigatus*.

L´amphotéricine B était historiquement le traitement de référence de l´infection aspergillaire. Cependant, des données plus récentes ont montré la supériorité du voriconazole par rapport à l´Amphotéricine. Le voriconazole est actuellement le traitement de première intention pour la plupart des formes d´aspergillose invasive. D´autres molécules telles que le posaconazole, l´itraconazole et l´isavuconazole sont des alternatives raisonnables. La durée optimale du traitement de l´ostéomyélite à *Aspergillus* n´est pas codifiée. Une durée d´au moins 6 à 8 semaines a été recommandée par les lignes directrices de la Société des Maladies infectieuses de l´Aspergillose de l´Amérique. Cependant une durée plus prolongée a été rapportée allant jusqu´à 8 mois [14]. Notre patient avait reçu 8 mois de traitement avec une évolution très favorable au plan clinique, biologique et radiologique.

## Conclusion

L´ostéomyélite aspergillaire doit être évoquée chez tout patient ayant une immunodépression devant l´évolution insidieuse, la négativité des prélèvements bactériens, la non réponse à un traitement présumé efficace et l´exposition au risque de transmission d´*Aspergillus*. La corrélation confirmée entre la contamination environnementale et l´incidence de l´aspergillose invasive nosocomiale incite au respect des mesures de prévention basées sur le contrôle de l´aérocontamination aspergillaire en milieu hospitalier.

## References

[1] Gabrielli E, Fothergill AW, Brescini L, Sutton DA, Marchionni E, Orsetti E (2014). Osteomyelitis caused by Aspergillus species: a review of 310 reported cases. ClinMicrobiol Infect.

[2] Aisner J, Schimpff SC, Bennett JE, Young VM, Wiernik PH (1976). Aspergillus infections in cancer patients: association with fire proofing materials in a new hospital. JAMA.

[3] Alberti C, Bouakline A, Ribaud P, Lacroix C, Rousselot P, Leblanc T (2001). Aspergillus study group: relationship between environmental fungal contamination and the incidence of invasive aspergillosis in haematology patients. J Hosp Infect.

[4] Frazier DD, Campbell DR, Garvey TA, Wiesel S, Bohlman HH, Eismont FJ (2001). Fungal infections of the spine: report of eleven patients with long term follow-up. J Bone Joint Surg Am.

[5] Walsh TJ, Anaissie EJ, Denning DW, Herbrecht R, Kontoyiannis DP, Marr KA (2008). Treatment of aspergillosis: clinical practice guidelines of the Infectious Diseases Society of America. Clinical Infectious Diseases.

[6] D´sa SR, Singh S, Satyendra S, Mathews P (2013). Case report of aspergillus osteomyelitis of the Ribs in an immunocompetent patient. J Glob Infect Dis.

[7] Asare KA, Jahng M, Pincus JL, Massie L, Lee SA (2013). Sternal osteomyelitis caused by Aspergillus fumigatus following cardiac surgery: case and review. Medical Mycology Case Reports.

[8] Derouin F, Alberti C, Bouakline A, Lacroix C, Ribaud P (2002). Corrélation entre la contamination fongique de l´environnement hospitalier et le risque d´aspergillose invasive nosocomiale. La Lettre de l´Infectiologue.

[9] Denning DW (1994). Invasive aspergillosis in immunocompromised patients. Curr Opin Infect Dis.

[10] Karia S, Jeyapalan K, Kennedy B (2011). Aspergillus fumigatus osteomyelitis in a patient receiving alemtuzumab for B-cell chronic lymphocytic leukaemia. Br J Haematol.

[11] Khemiri M, El fekih N, Borgi A, Kharfi M, Boubaker S, Barsaoui S (2012). Pseudotumoral cutaneous aspergillosis in chronic granulomatous disease: report of a pediatric case. Am J Dermatopathol.

[12] Yu OH, Keet AW, Sheppard DC, Brewer T (2010). Articular aspergillosis: case report and review of the literature. Int J Infect Dis.

[13] Zhu LP, Chen XS, Wu JQ, Yang FF, Weng XH (2011). Aspergillus vertebral osteomyelitis and ureteral obstruction after liver transplantation. Transpl Infect Dis.

[14] Landaburu MF, López Daneri G, Ploszaj F, Kruss M, Vinante A, Vecino CH (2019). Osteomyelitis of the rib cage by Aspergillus flavus. Rev Iberoam Micol.

